# Compliance with the guidelines on recommended immunization schedule in patients with inflammatory bowel disease: implications on public health policies

**DOI:** 10.1186/s12889-020-08850-y

**Published:** 2020-05-19

**Authors:** Cristina García-Serrano, Glòria Mirada, Josep R Marsal, Marta Ortega, Joaquim Sol, Rubén Solano, Eva M Artigues, Pepi Estany

**Affiliations:** 1 Catalan Health Institute (ICS), Primary Care, Lleida, Spain; 2 Lleida Institute for Biomedical Research (IRBLleida), Lleida, Spain; 3 Faculty of Nursery and Physiotherapy, University of Lleida, Lleida, Spain; 4 Catalan Agency of Public Health, Lleida, Spain; 5 Cardiovascular Epidemiology Unit, Cardiology Department, Vall d’Hebron University Hospital, CIBERESP, Lleida, Spain; 6 Research Support Unit Lleida, Fundació Institut Universitari per a la recerca a l’Atenció Primària de Salut Jordi Gol i Gurina (IDIAPJGol), Lleida, Spain; 7 Faculty of Medicine, University of Lleida, Lleida, Spain; 8 Research Group in Therapies in Primary Care (GRETAPS), Lleida, Spain; 9 Metabolic Physiopathology Group, Department of Experimental Medicine, University of Lleida-IRBLleida, Lleida, Spain; 10 Research Group in Health Education (GREpS), Department of Nursery and Physiotherapy, University of Lleida, Lleida, Spain

**Keywords:** Inflammatory bowel disease, Epidemiology, Immunization schedule, Public health

## Abstract

**Background:**

Patients with inflammatory bowel disease (IBD) have a higher risk of developing opportunistic infections due to either the disease itself or to treatment with immunosuppressants. This risk can be reduced through vaccination. The aim of this study was to determine the prevalence of compliance with the guidelines on recommended immunization schedule in patients with IBD in the health district of Lleida, Spain.

**Methods:**

Descriptive, cross-sectional, retrospective study of data at December 31, 2016. The reference population was formed by adults with a clinical diagnosis of IBD. The dependent variable was “compliance with the guidelines on recommended immunization schedule”. Variables were sex, age, residence, diagnosis, vaccination against measles, mumps, rubella, varicella, tetanus-diphtheria, influenza, pneumococcus, meningococcus C, hepatitis B, and hepatitis A. Data were obtained from electronic medical records. For the data analysis, mean (standard deviation), prevalence with 95% confidence intervals, χ^2^ test and Mann-Whitney test were used.

**Results:**

Compliance did not exceed 65% for any of vaccines analysed in the 1722 studied patients with ulcerative colitis or Crohn’s disease. Significant differences across age groups were found in compliance for measles, mumps, rubella, varicella, tetanus, diphtheria and influenza in both ulcerative colitis and Crohn’s disease and for meningococcus C and hepatitis A exclusively in ulcerative colitis.

**Conclusions:**

Compliance in patients with IBD is low. Thus, prevention of immunopreventable diseases or their complications is not maximized in this kind of patients. Greater awareness of how vaccines can reduce the risk of vaccine-preventable infections is needed among both patients and healthcare professionals.

## Background

Ulcerative colitis (UC) and Crohn’s disease (CD) are subcategories of inflammatory bowel disease (IBD) [1–3]. They constitute a chronic inflammatory condition that affects the gastrointestinal tract and is caused by altered immune responses against gut microbiota. Possible triggers include diet, virus exposure, smoking, psychosocial stress, and other factors that alter the pathogenesis of IBD [2, 3]. It generates high direct health costs associated with hospitalization, surgery, pharmacological treatment, and check-ups starting in the third year after diagnosis. Hospitalization costs alone have been found to account for 50 to 80% of all direct health costs [1].

Over 1.5 million individuals in North America and 2 million in Europe are estimated to have IBD [4]. The reported prevalence in Spain is 205 cases per 100,000 inhabitants [5], although the accuracy of estimates in Europe is limited by small sample sizes. To address this problem in Spain, a nationwide prospective population-based cohort study known as *EpidemIBD* was launched in 2017 to determine the true epidemiological scale of IBD [6].

Recent years have seen an increase in the number of IBD patients who need treatment with immunosuppressive agents such as thiopurines (azathioprine, mercaptopurine), methotrexate, calcineurin inhibitors (ciclosporin, tacrolimus), biologics (infliximab, adalimumab), and corticosteroids (prednisolone or equivalent at a dose of ≥20 mg for at least 2 weeks) [7–9]. These patients are considered to be immunosuppressed [1, 10, 11] and predisposed to a higher risk of opportunistic infections [9], as evidenced by numerous case reports and series [1, 7, 12, 13]. Treatment with immunosuppressive agents has been associated with a 3.9-fold increased risk for opportunistic infections in patients with IBD, with further analysis showing a 2.9-fold increased risk for the use of any one agent and a 14.5-fold increased risk for the use of two or three agents [7]. Opportunistic infections are frequently associated with significant morbidity and mortality and may also result in reduced treatment effectiveness [8, 9]. Patients at risk, however, can be protected through vaccination [7, 14–18]. Several guidelines are available to support clinical practice, including a 2010 practical guide to vaccinate patients with IBD [17] and recommendations from working groups such as the Spanish Society of Preventive Medicine, Public Health, and Hygiene [18] and the European Crohn’s and Colitis Organisation [11]. The vaccination manual of the Catalan Public Health Agency did not include a specific section devoted to the vaccination of patients with IBD until 2018 [19].

Evidence on the immunogenicity and safety of vaccines in treated patients with IBD is still limited, as different immunomodulators can alter immune responses to vaccines [7, 11, 15]. According to some studies, patients with IBD had lower antibody responses after hepatitis B vaccination than the general population [7, 20, 21]. In other studies, IBD patients under treatment with infliximab and immunomodulatory therapy showed an impaired response to a single dose of trivalent inactivated influenza vaccine [22, 23]. For this reason, and due to their increased risk of opportunistic infections in the first year of immunosuppressive therapy, IBD patients should be vaccinated as soon as possible after diagnosis [1, 15, 24, 25]. Prevention of infectious disease in this population is a public health issue and vaccination may be an effective tool.

In the sanitary region of Lleida, Spain, IBD patients are prescribed biologics and monitored in specialist care settings, but vaccines are routinely administered in primary care and recorded in a centralized electronic database. Although the access to vaccination is free and universal in Spain and many infections can be prevented by vaccination, coverage in clinical practice remains uncertain. Better communication between practitioners working at different levels of care is essential to prevent under-recording and under-immunization [1, 25].

This study represented the first step towards optimal vaccination coverage, and aimed to determine the proportion of IBD patients who have been appropriately vaccinated according to the recommended immunization schedule in the health district of Lleida, Spain.

## Methods

### Aim

The aim of this study was to determine the proportion of IBD patients who have been appropriately vaccinated according to the recommended immunization schedule in the health district of Lleida, Spain in order to settle the basis for a future intervention in both primary care and hospitals to reach a better compliance of these patients.

### Study design and setting, data collection and information sources

Retrospective, cross-sectional descriptive analysis of data from the Catalan Health Institute’s ECAP database. Records introduced in the database until December 31, 2016 corresponding to all the eligible patients assigned to a primary care unit in the health district of Lleida, Spain were obtained.

ECAP database was fully implemented in all Lleida primary care settings in 2005, but records collected in physical format before implementation were introduced in the database between 1998 and 2005. This database is routinely used by all primary care practitioners to collect electronic medical records from the patients, including administrative data, medical conditions, vaccinations, prescriptions and laboratory results, as well as diagnosis associated with hospital and outpatient visits, attended by the Catalan primary public healthcare system.

### Reference and study population

The reference population was formed by adults with IBD in the healthcare district of Lleida. To be included, patients had to have a clinical diagnosis of UC or CD, be 18 years or older, and be assigned to a primary care unit in the sanitary district of Lleida. In order to focus the study on the immunization that depended directly on the health system, patients with a history of an allergic reaction to a vaccine or to any component of a vaccine needed to achieve adequate immunization were excluded, as were seronegative patients who had refused to be vaccinated.

## Variables

The dependent variable was “compliance with the guidelines on recommended immunization schedule”, from now on “compliance” (yes/no), which was assessed according to compliance with vaccination dosing and schedule recommendations for the disease in question (UC or CD), immunosuppression status, and serology results [26, 27] (Table 1). The above information was entered into a purpose-designed algorithm applied to each patient to determine whether or not they were adequately immunized.

**Table 1:** Criteria to assess compliance

**Infections**	**Identifying events** **(at least one of the following criteria should be met)**
Measles, mumps and rubella	Date of birth before January 01, 1966 History of measles, mumps, rubella Record of seropositivity for all three diseases: measles IgG level > 16.5 IU/mL; mumpsIgGlevel11 IU/mL, rubella IgG level > 15 IU/mL Record of two MMR vaccine doses administered at least 1 month apart
Varicella	History of varicella Record of seropositivity for varicella zoster virus: IgG level > 165 IU/mL Record of two varicella vaccine doses administered at least 1 month apart
Tetanus	Record of at least five doses of tetanus toxoid vaccine administered before the age of 16 years Record of at least three doses of tetanus toxoid vaccine administered at a minimum interval of 0–1-6 months after the age of 7 years
Influenza	Record of influenza vaccine administration: high-dose seasonal influenza vaccine for patients aged > 60 or > 65 years or standard influenza vaccine for other patients
Pneumococcus	Record of one dose of 13-valent pneumococcal conjugate vaccine (PCV13) and one dose of pneumococcal polysaccharide vaccine (PPSV23) administered at least 2 months apart Record of one dose of PCV13 and one dose of PPSV23 administered at least 12 months apart Record of at least one dose of PCV13 administered in the past year Record of at least one dose of PPSV23 administered in the past year
Meningococcus C	Record of at least one dose of meningococcal vaccine administered after 12 months of age
Hepatitis B	History of hepatitis B and record of seropositivity for hepatitis B surface antigen (HBsAg) > 0.9 IU/mL; total antibodies (anti-HBc) > 0.9 IU/mL Post-vaccine record of seropositivity for anti-HBs > 12 IU/mL^a^ Record of three doses of hepatitis B vaccine administered at a minimum interval of 0–1-6 months Record of four doses of hepatitis B vaccine administered at a minimum interval of 0–1–2-6 months Record of three doses of hepatitis A + B vaccine administered at a minimum interval of 0–1-6 months
Hepatitis A	History of hepatitis A Record of seropositivity: IgG level > 40 IU/mL Record of two doses of monovalent vaccine administered at least 6 months apart Record of three doses of combined hepatitis A + B vaccine administered at a minimum interval of 0–1-6 months.

*IgG* immunoglobulin G

^a^According to laboratory criteria of the health district of Lleida, Spain

The independent variables were sex, age, area of residence (rural vs. urban), diagnosis (CD or UC), and primovaccination against measles, mumps, and rubella (MMR), varicella, tetanus-diphtheria, influenza, pneumococcus, meningococcus C, hepatitis B, and hepatitis A [26, 27].

### Statistical analysis

Quantitative variables were expressed as mean and standard deviation (SD) and qualitative variables as percentages. Prevalence estimates were presented with 95% confidence intervals. Differences between compliance rates according to the study variables were analysed using the χ^2^ or Mann-Whitney test as appropriate. Statistical significance was established at a *p* value < 0.05.

## Results

In total, 1722 patients were studied: 1420 (82.5%) had UC and 302 (17.5%) had CD. Patients with UC had a mean (SD) age of 54.6 (18.8) years, with an age range from 18 to 97, 50.8% were women, and 66.4% lived in a rural area. Those with CD had a mean (SD) age of 47.8 (16.6) years, with an age range from 18 to 96, 44.7% were women, and 63.9% lived in a rural area (Table 2). Compliance did not exceed 65% in any of the two diseases. Significant differences in compliance for all the studied vaccines except for MMR were observed between patients with UC and CD, with higher rates in CD except for vaccination against influenza (Table 3). Cause of compliance (vaccination or natural immunity) is specified in the Supplementary table.

**Table 2:** Sociodemographic characteristics, patients with ulcerative colitis and Crohn’s disease, sanitary district of Lleida (Spain) 2016

	Ulcerative colitis No. (%)	Crohn’s disease No. (%)	*P* Value
No. of patients	1420	302	
Sex (female)	721 (50.8%)	135 (44.7%)	0.064
Rural area (yes)	943 (66.4%)	193 (63.9%)	0.444
Age (SD), y	54.6 (18.8)	47.8 (16.6)	< 0.001

**Table 3:** Compliance in patients with ulcerative colitis and Crohn’s disease, sanitary district of Lleida (Spain) 2016

Infections	Ulcerative colitis No. (%) (95% CI)	Crohn’s disease No. (%) (95% CI)	*P* value
Measles, mumps, rubella	912 (64.2%) (61.7–66.7)	177 (58.6%) (52.8–64.2)	0.076
Varicella	335 (23.6%) (21.4–25.8)	134 (44.4%) (38.8–50.0)	< 0.001
Tetanus-diphtheria	391 (27.5%) (25.2–29.9)	121 (40.1%) (34.5–45.6)	< 0.001
Influenza	386 (27.2%) (24.9–29.5)	45 (14.9%) (10.9–18.9)	< 0.001
Pneumococcus	96 (6.8%) (5.5–8.1)	58 (19.2%) (14.8–23.6)	< 0.001
Meningococcus C	111 (7.8%) (6.4–9.2)	71 (23.5%) (18.7–28.3)	< 0.001
Hepatitis B	99 (7%) (5.6–8.3)	69 (22.8%) (18.1–27.6)	< 0.001
Hepatitis A	27 (1.9%) (1.2–2.6)	14 (4.6%) (2.3–7.0)	0.009

*CI* Confidence interval

Significant differences between age groups were found in compliance for MMR, varicella, tetanus, diphtheria and influenza in both UC and CD (Table 4, Table 5), and for meningococcus C and hepatitis A exclusively in UC (Table 5). Compliances for MMR and seasonal influenza among patients with UC or CD were associated to higher age groups (Table 4, Table 5).

**Table 4:** Compliance by age in patients with Crohn’s disease, sanitary district of Lleida (Spain) 2016

Infections	≤ 40 y (*n* = 114)	41–60 y (*n* = 127)	≥ 61 y (*n* = 61)	Total (*n* = 302)	*P* value
	No. (%) (95% CI)	No. (%) (95% CI)	No. (%) (95% CI)	No. (%) (95% CI)	
Measles, mumps, rubella	30 (26.3%) (18.2–4.4)	86 (67.7%) (58.8–75.7)	61 (100%) (94.1–100)	177 (58.6%) (52.8–64.2)	< 0.001
Varicella	60 (52.6%) (43.5–61.8)	55 (43.3%) (34.7–51.9)	19 (31.1%) (19.5–42.8)	134 (44.4%) (38.8–50.0)	0.006
Tetanus and diphtheria	35 (30.7%) (22.2–39.2)	57 (44.9%) (36.2–53.5)	29 (47.5%) (35.0–60.1)	121 (40.1%) (34.5–45.6)	0.016
Influenza	1 (0.9%) (0.0–2.6)	0 (0%) (0.0–0.0)	44 (72.1%) (60.9–83.4)	45 (14.9%) (10.9–18.9)	< 0.001
Pneumococcus	19 (16.7%) (9.8–23.5)	27 (21.3%) (14.4–28.4)	12 (19.7%) (9.7–29.6)	58 (19.2%) (14.8–23.6)	0.531
Meningococcus C	24 (21.1%) (13.6–28.5)	37 (29.1%) (21.2–37.0)	10 (16.4%) (7.1–25.7)	71 (23.5%) (18.7–28.3)	0.778
Hepatitis B	19 (16.7%) (9.8–23.5)	35 (27.6%) (19.8–35.3)	15 (24.6%) (13.8–35.4)	69 (22.8%) (18.1–27.6)	0.134
Hepatitis A	8 (7%) (2.3–11.7)	5 (3.9%) (0.6–7.3)	1 (1.6%) (0.0–4.8)	14 (4.6%) (2.3–7.0)	0.094

*CI* Confidence interval.

**Table 5:** Compliance by age group in patients with ulcerative colitis, sanitary district of Lleida (Spain) 2016

Infections	≤ 40 y (*n* = 377)	41–60 y (*n* = 511)	≥ 61 y (*n* = 532)	Total (*n* = 1420)	*P* value
	No. (%) (95% CI)	No. (%) (95% CI)	No. (%) (95% CI)	No. (%) (95% CI)	
Measles, mumps, rubella	60 (15.9%) (12.2–19.6)	320 (62.6%) (58.3–66.8)	532 (100%) (99.3–100)	912 (64.2%) (61.7–66.7)	< 0.001
Varicella	136 (36.1%) (31.2–40.9)	147 (28.8%) (12.2–19.6)	52 (9.8%) (7.2–12.3)	335 (23.6%) (21.4–25.8)	< 0.001
Tetanus-diphtheria	61 (16.2%) (12.5–19.9)	152 (29.7%) (25.8–33.7)	178 (33.5%) (29.4–37.5)	391 (27.5%) (25.2–29.9)	< 0.001
Influenza	1 (0.3%) (0.0–0.8)	7 (1.4%) (0.4–2.4)	378 (71.1%) (67.2–74.9)	386 (27.2%) (24.9–29.5)	< 0.001
Pneumococcus	21 (5.6%) (3.3–7.9)	36 (7%) (4.8–9.3)	39 (7.3%) (5.1–9.5)	96 (6.8%) (5.5–8.1)	0.316
Meningococcus C	35 (9.3%) (6.4–12.2)	55 (10.8%) (8.1–13.5%)	21 (3.9%) (2.3–5.6%)	111 (7.8%) (6.4–9.2%)	0.001
Hepatitis B	19 (5%) (2.8–7.2%)	56 (11%) (8.3–13.7)	24 (4.5%) (2.7–6.3)	99 (7%) (5.6–8.3)	0.445
Hepatitis A	14 (3.7%) (1.8–5.6)	12 (2.3%) (1.0–3.7)	1 (0.2%) (0.0–0.6)	27 (1.9%) (1.2–2.6)	< 0.001

*CI* Confidence interval.

## Discussion

IBD is associated with high morbidity and premature death, and represents a high financial burden in the form of direct costs (e.g., hospitalization, treatment, check-ups), indirect costs (e.g., productivity loss and disability), and intangible costs (e.g., loss of quality of life) [1]. Furthermore, patients diagnosed with IBD are at higher risk of suffering opportunistic infections [9, 15, 28, 29] and at the same time, of a higher severity of these infections [8, 29]. Most of these opportunistic infections are preventable with adequate vaccination according to underlying pathology [8, 10, 15, 24], for this reason, vaccination regardless of age against measles, mumps, rubella, varicella, tetanus and diphtheria, pneumococcus and influenza is recommended [26, 27]. The low compliance rates in patients with IBD observed in our study, added to the reported or disputed alteration of immunogenicity of vaccines in this kind of patients [8, 10, 23, 30] implies a public health problem that must be taken into account in order to look for improvement strategies.

Latest official data regarding seasonal influenza vaccination coverage in the Catalan population aged 60 years or older reported a coverage of 47.7% [31]. In 2016–2017, a vaccination coverage of 54.3% was reported for population aged 65 years or older [32]. In our study, we observe coverage rates for seasonal influenza of more than 70% in patients aged 61 years or older. However, these rates decrease to nearly zero in younger groups, suggesting that patients would have been recruited for vaccination according to seasonal campaigns aimed to aged population rather than according to their specific risk group. This possibility is supported by the findings of a Spanish study on pneumococcal vaccination coverage, which found higher uptake in patients for whom routine vaccination was indicated (generally older adults), regardless of their risk profile [33]. Similarly, the high tetanus-diphtheria compliance observed in our series could be due to the inclusion of these vaccines in routine vaccination programs.

Vaccination against varicella has been recommended for all immunocompromised seronegative patients with IBD [8, 10, 15], and although seroprevalence against varicella in Spain is estimated to be around 93% [34], seronegative patients must be detected and vaccinated in order to assure adequate immunization. Thus, the low compliance observed for varicella vaccination in our series is worrying, especially because of its risk of complication to herpes zoster at adult age, which is higher in immunosuppressed patients, and particularly in those receiving immunosupressive therapy [15, 35]. However, vaccination for varicella is contraindicated in patients already on immunosuppressive therapy due to the increased risk of herpes zoster infection [10, 15, 28]. Thus, it is important, if possible, to vaccinate before initiation of therapy and to ensure that cohabitants of IBD patients are protected against this disease [10, 15, 19].

Hepatitis B compliance is highest in patients aged between 41 and 60 years but overall compliance is low. Other authors have reported low response rates to hepatitis B vaccine in patients with IBD, particularly in those on immunosuppressive therapy and with active disease [36], resulting in inadequate hepatitis B surface antibody levels in 70% of vaccinated patients [5]. In this sense, vaccination during remission or periods of adequate immune function has been recommended [1, 8, 15, 36].

The present study shows that compliance is better in patients with CD for all diseases except for MMR and influenza, these two latter cases because of the higher proportion of older patients in UC. Other authors have associated disease severity to higher adherence to treatment [37]. In this line, we hypothesize that disease severity, added to the higher risk for opportunistic infections of CD compared to UC patients [8], could also be associated to the higher compliance due to the higher awareness of both patients and practitioners about their condition. However, compliance is low, and does not exceed 65% for any of the vaccines analysed. This observation coincides with the findings of several authors, who have reported suboptimal coverage levels in both primary care and hospital settings [15, 25, 38].

In the sanitary district of Lleida most patients with IBD are diagnosed in hospital settings, but vaccines are mainly administered by primary care practitioners and records are kept in the centralized ECAP database. Sometimes, patients do not visit their primary care centre for vaccination, and in other cases, insufficient or confusing information regarding their health status or their treatment is provided: approximately 50% of patients with IBD do not know that they need to be vaccinated against certain diseases [25]. This lack of awareness, together with other barriers that complicate compliance such as vaccination costs and incorrect or inadequate vaccination recommendations by healthcare professionals, must be addressed [25, 39].

Interventions involving patient education and continuous training for healthcare professionals are needed to improve compliance among IBD patients [7, 17, 25, 40], and standardized vaccination protocols are therefore essential for reducing vaccine-preventable infections and improving patient quality of life. In this sense, in 2018 the Spanish Ministry of Health, Consumption, and Social Wellbeing published *vaccination recommendations for patients of all ages at risk of vaccine-preventable diseases*, which included specific protocols for IBD [41]. Furthermore, several authors have proposed using online systems to facilitate coordination between professionals working at different levels of care [1, 25]. Options include the use of electronic devices, electronic reminders for both patients and healthcare professionals, and pop-up checklists. In one study, an automatic computer vaccination reminder system for patients under treatment with infliximab led to higher uptake rates [25].

The observed low compliance is a public health issue in the studied health district, and according to the commented recommendations, several actions that could increase compliance have been proposed and are already being implemented. These actions include continuous professional development programs for healthcare professionals, online meetings with regional vaccination managers and implementation of telematics, online and other systems to improve communication between different levels of care. A connection between ECAP database and hospital software that updates all relevant information such as vaccines has been required to the database managers.

Our study has some limitations. As our findings are based on a retrospective review of data from the ECAP database, they may be subject to information bias in the form of incomplete or missing data on vaccinations, diagnoses, and analysis results. Incomplete records could be aggravated because of the different databases used in primary care respect to hospital services, as well as the impossibility of access to private care databases. For this reason, and although most of the patients are attended in the public health services, a uniform database containing data from all the mentioned sources is essential to obtain more reliable data. Nevertheless, these missing records would also be found in daily practice, and compliance was determined the same way as it would have been in clinical practice, so all the ECAP records were carefully analysed to minimize the risk of information bias. Because of the difficulty of defining immunosuppression in a cross-sectional study, this study did not include data regarding immunosuppressive medication, so another limitation is that we were not able to discern between immunosuppressed and non immunosuppressed patients. We expect to dispose of this data in further studies.

## Conclusions

Compliance with the guidelines of the recommended vaccination schedule in patients with IBD is low. Thus, prevention of immunopreventable diseases or their complications is not maximized in this kind of patients. Awareness about the need for vaccination in patients with IBD must also be improved among both patients and healthcare professionals, as immune responses can be impaired by the disease itself and the use of immunosuppressive therapy. We propose to unify electronic health records between primary care and hospital settings by creating efficient communication and warning systems. Efficiency and safety in clinical practice and control of pathologies would be maximized if this database also included information from private care and other relevant instances. It would be interesting to conduct follow-up studies to determine the incidence of complications in unvaccinated patients that could have been prevented through vaccination.

## Supplementary information

**Additional file 1.** Supplementary table. Cause of compliance by age. Sanitary district of Lleida (Spain) 2016

## Data Availability

The datasets used and/or analysed during the current study are available from the corresponding author on reasonable request.

## Abbreviations

CI: Confidence interval
IBD: Inflammatory bowel disease
Ig: Immunoglobulin
MMR: Measles, mumps, rubella
PCV13: 13-valent pneumococcal conjugate vaccine
PPSV23: Pneumococcal polysaccharide vaccine
SD: Standard deviation
