# Supplementary material for: Compliance with the guidelines on recommended immunization schedule in patients with inflammatory bowel disease: implications on public health policies
Source: BMC Public Health. 2020 May 19;20:713. doi: 10.1186/s12889-020-08850-y (PMC7236120; doi:10.1186/s12889-020-08850-y)
Supplement: Supplementary file 1 — Additional file 1. Supplementary table. Cause of compliance by age. Sanitary district of Lleida (Spain) 2016 [file 12889_2020_8850_MOESM1_ESM.docx]

| **Supplementary table. Cause of compliance by age. sanitary district of Lleida (Spain) 2016** | | | | | | | | | |
| --- | --- | --- | --- | --- | --- | --- | --- | --- | --- |
|  |  | Ulcerative Cholitis | | | | Chron's disease | | | |
| Infections | Cause of compliance | **≤ 40 y** | **41-60 y** | **≥ 61 y** | **Total** | **≤ 40 y** | **41-60 y** | **≥ 61 y** | **Total** |
|  |  | (n = 377) | (n = 511) | (n = 532) | (n = 1420) | (n = 114) | (n = 127) | (n = 61) | (n = 302) |
| Measles,mumps, rubella | Total | 60 (100%) | 320 (100%) | 532 (100%) | 912 (100%) | 30 (100%) | 86 (100%) | 61 (100%) | 177 (100%) |
|  | Vaccinated | 16 (26.7%) | 10 (3.1%) | 0 (0%) | 26 (2.9%) | 11 (36.7%) | 2 (2.3%) | 0 (0%) | 13 (7.3%) |
|  | Register of disease/positive serology | 44 (73.3%) | 310 (96.9%) | 532 (100%) | 886 (97.1%) | 19 (63.3%) | 84 (97.7%) | 61 (100%) | 164 (92.7%) |
| Varicella | Total | 136 (100%) | 147 (100%) | 52 (100%) | 335 (100%) | 60 (100%) | 55 (100%) | 19 (100%) | 134 (100%) |
|  | Vaccinated | 2 (1.5%) | 1 (0.7%) | 0 (0%) | 3 (0.9%) | 1 (1.7%) | 0 (0%) | 0 (0%) | 1 (0.7%) |
|  | Register of disease/positive serology | 134 (98.5%) | 146 (99.3%) | 52 (100%) | 332 (99.1%) | 59 (98.3%) | 55 (100%) | 19 (100%) | 133 (99.3%) |
| Hepatitis B | Total | 19 (100%) | 56 (100%) | 24 (100%) | 99 (100%) | 19 (100%) | 35 (100%) | 15 (100%) | 69 (100%) |
|  | Vaccinated | 15 (78.9%) | 36 (64.3%) | 12 (50%) | 63 (63.6%) | 11 (57.9%) | 11 (31.4%) | 9 (60%) | 31 (44.9%) |
|  | Register of disease/positive serology | 4 (21.1%) | 20 (35.7%) | 12 (50%) | 36 (36.4%) | 8 (42.1%) | 24 (68.6%) | 6 (40%) | 38 (55.1%) |
| Hepatitis A | Total | 14 (100%) | 12 (100%) | 1 (100%) | 27 (100%) | 8 (100%) | 5 (100%) | 1 (100%) | 14 (100%) |
|  | Vaccinated | 3 (21.4%) | 0 (0%) | 0 (0%) | 3 (11.1%) | 0 (0%) | 0 (0%) | 0 (0%) | 0 (0%) |
|  | Register of disease/positive serology | 11 (78.6%) | 12 (100%) | 1 (100%) | 24 (88.9%) | 8 (100%) | 5 (100%) | 1 (100%) | 14 (100%) |
